# Research Progress on Varicella-Zoster Virus Vaccines

**DOI:** 10.3390/vaccines13070730

**Published:** 2025-07-04

**Authors:** Hongjing Liu, Lingyan Cui, Sibo Zhang, Hong Wang, Wenhui Xue, Hai Li, Yuyun Zhang, Lin Chen, Ying Gu, Tingting Li, Ningshao Xia, Shaowei Li

**Affiliations:** 1 State Key Laboratory of Vaccines for Infectious Diseases, Xiang An Biomedicine Laboratory, School of Public Health, School of Life Sciences, Xiamen University, Xiamen 361102, China; 2 National Institute of Diagnostics and Vaccine Development in Infectious Diseases, State Key Laboratory of Molecular Vaccinology and Molecular Diagnostics, Collaborative Innovation Center of Biologic Products, National Innovation Platform for Industry-Education Integration in Vaccine Research, Xiamen University, Xiamen 361102, China

**Keywords:** varicella-zoster virus, varicella, herpes zoster, vaccines, cell-mediated immunity, vaccine platforms

## Abstract

Varicella-zoster virus (VZV) poses significant public health challenges as the etiological agent of varicella (chickenpox) and herpes zoster (HZ), given its high transmissibility and potential for severe complications. The introduction of VZV vaccines—particularly the vOka-based live attenuated and glycoprotein gE-based recombinant subunit vaccines—has substantially reduced the global incidence of these diseases. However, live attenuated vaccines raise concerns regarding safety and immunogenicity, especially in immunocompromised populations, while recombinant subunit vaccines, such as Shingrix, exhibit high efficacy but are associated with side effects and adjuvant limitations. Recent advancements in vaccine technology, including mRNA vaccines, viral vector vaccines, and virus-like particle (VLP) vaccines, offer promising alternatives with improved safety profiles and durable immunity. This review synthesizes current knowledge on VZV vaccine mechanisms, clinical applications, and immunization strategies, while also examining future directions in vaccine development. The findings underscore the pivotal role of VZV vaccines in disease prevention and highlight the need for continued research to enhance their public health impact.

## 1. Introduction

**Figure 1:**
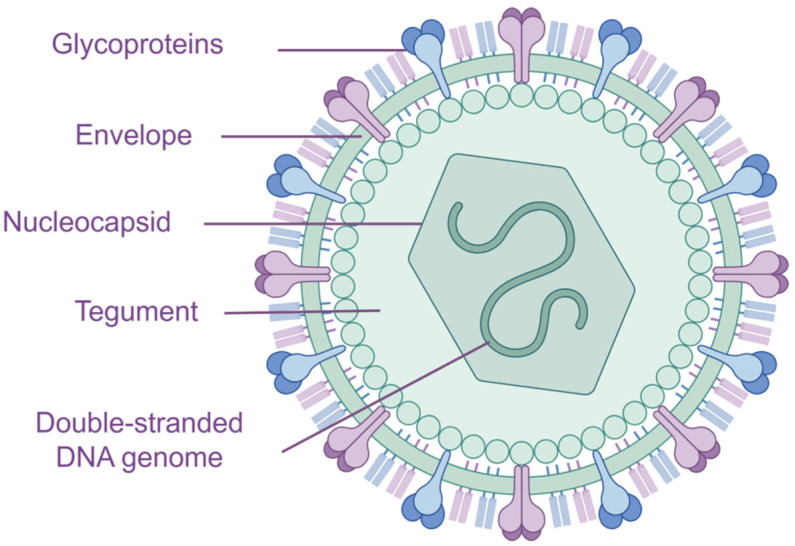
Structure of VZV. The varicella-zoster virus (VZV) virion is nearly spherical, with a diameter of approximately 150–200 nm. It consists of a double-stranded DNA genome, a nucleocapsid, and a lipoprotein envelope. The nucleocapsid is composed of 162 capsomeres arranged in an icosahedral structure, while the envelope is densely studded with surface glycoproteins, which play crucial roles in viral entry and immune evasion. [Source: https://www.figdraw.com/].

Varicella-zoster virus (VZV), also known as human herpesvirus 3 (HHV-3), belongs to the *Herpesviridae* family and the *Alphaherpesvirinae* subfamily. This nearly spherical, enveloped virus measures 150–200 nm in diameter and contains double-stranded DNA. Its structural components include nucleocapsid proteins and a lipoprotein envelope (Figure 1). The VZV genome consists of 124,884 base pairs (bp) and encodes at least 70 genes, organized into two covalently linked segments: a long (L) segment and a short (S) segment [1]. The nucleocapsid forms an icosahedral structure with 162 capsomeres, while the viral envelope is enriched with glycoproteins, including gB (ORF31), gC (ORF14), gE (ORF68), gH (ORF37), gI (ORF67), gK (ORF5), gL (ORF60), gM (ORF50), and gN (ORF50) [2]. These glycoproteins play vital roles in the viral life cycle and interactions with the immune system.

VZV is a highly contagious pathogen that spreads through aerosol particles or direct contact with vesicular lesions of infected individuals. Primary VZV infection causes varicella (chickenpox), which predominantly occurs in children and manifests as fever, fatigue, and an intensely pruritic vesicular rash, typically following seasonal patterns [3]. After primary infection, VZV becomes latent in dorsal root ganglia and can reactivate later in life due to immune decline or immunosenescence, leading to herpes zoster (HZ, shingles). Although HZ is less contagious than varicella, exposure to VZV from the vesicular lesions of HZ patients can still induce varicella in susceptible individuals [4].

While varicella and HZ are typically self-limiting, they may lead to severe or fatal complications. Varicella can induce secondary bacterial infections, encephalitis, pneumonia, hepatitis, hemorrhage, and other life-threatening conditions. Similarly, HZ is frequently complicated by postherpetic neuralgia (PHN), as well as meningitis, meningoencephalitis, meningoradiculitis, cerebellitis, myelopathy, vasculopathy, and ophthalmic complications [5], all of which seriously compromise of patients’ quality of life [6,7,8,9]. Among immunocompromised individuals, VZV infection may result in more severe complications while enhancing viral transmissibility, thereby exacerbating disease incidence and mortality rates [10,11]. Varicella, HZ and their complications cause significant productivity losses and impose a substantial burden on healthcare systems.

Currently, vaccination remains the primary strategy for preventing varicella and HZ. Before widespread varicella vaccination (1996–2011), approximately 4 million cases of varicella occurred annually in the United States, resulting in an estimated 10,000 hospitalizations and 100 deaths each year [12]. Studies indicate that implementation of a two-dose varicella vaccination program achieved a 95% reduction in varicella incidence in the United States and an 84% decrease in varicella-related hospitalizations among Australian children aged 18–59 months [13,14]. However, unvaccinated adults or those without prior varicella exposure remain susceptible to infection. Global epidemiological data indicate an annual HZ incidence of 3–5 cases per 1000 individuals [15]. The live zoster vaccine (Zostavax) and recombinant adjuvanted vaccine (Shingrix) were approved by the Food and Drug Administration (FDA). However, with global aging populations, HZ incidence is projected to increase, imposing growing economic burdens on healthcare systems. Additionally, immunocompromised populations, including cancer patients, organ transplant recipients, and human immunodeficiency virus (HIV)-positive individuals, face particular challenges with varicella and HZ vaccines due to impaired immune responses against VZV.

This review systematically outlines recent advancements in the development of VZV vaccines for both varicella and HZ. We conducted a comprehensive literature search using PubMed, supplemented by manual searches from Google Scholar. Vaccine-related data were sourced from official pharmaceutical company websites, vaccine package inserts, published literature, the Center for Drug Evaluation (CDE) of China’s National Medical Products Administration (NMPA) (https://www.cde.org.cn/), and ClinicalTrials.gov (https://clinicaltrials.gov/). Through a comprehensive analysis of both established and emerging vaccine strategies, this study aims to provide a robust scientific foundation and strategic guidance for future research in VZV vaccines.

## 2. Varicella Vaccines

Following VZV infection, the humoral immune system generates virus-specific antibodies capable of neutralizing the virus and blocking its entry into host cells, thereby limiting viral dissemination [16]. These antibodies exhibit long-term persistence, conferring durable immunity and substantially lowering the risk of reinfection. Additionally, VZV infection triggers a robust cellular immune response, mediated by CD4+ and CD8+ T-cells that recognize and eliminate infected cells, which plays a critical role in preventing viral reactivation [17]. The varicella vaccine effectively stimulates both humoral and cellular immunity against VZV, enabling control of primary infection while potentially reducing the incidence of HZ.

Varicella vaccines are formulated using live attenuated varicella virus strains and are available in two primary forms: a monovalent varicella vaccine and a measles–mumps–rubella–varicella (MMRV) combination vaccine (Table 1). MMRV confers protection against varicella along with three other common pediatric infectious diseases. Both vaccine formulations have demonstrated efficacy and safety in extensive global epidemiological studies and clinical trials.

### 2.1. Monovalent Varicella Vaccines

Currently, commercially available varicella vaccines are predominantly based on the vOka strain of live attenuated varicella virus. The vOka strain was derived from the parental Oka strain (pOka) of VZV, which was isolated in 1974 by Takahashi et al. from a child with varicella and subsequently attenuated through serial passaging in human embryonic lung (HEL) cells, guinea pig embryo (GPE) cells, and human diploid (WI-38) cells [31]. Experimental evidence shows that vOka maintains stable immunogenicity through at least 15 passages in WI-38 cells, supporting its suitability for large-scale vaccine production [32]. While the vOka vaccine contains a heterogeneous population of attenuated live viruses with minor inter-manufacturer and inter-batch variations, these differences are clinically insignificant [33]. Notably, the vOka strain demonstrates remarkable genetic stability, a critical attribute for vaccine safety. Compared to wild-type VZV, vOka induces a milder immune response while maintaining robust protective immunity and significantly lowering the incidence of HZ [34]. Large-scale clinical trials conducted in diverse age groups and populations with varying health statuses have confirmed the safety, efficacy, and long-term protective effects of vOka-based varicella vaccines [28,35,36,37].

Japan pioneered the use of the vOka varicella vaccine, granting approval to OkaVax (Biken) in 1984, marking the first national licensure of this vaccine. Subsequently, numerous countries have adopted varicella vaccination into their national immunization programs [18]. In 1995, the FDA approved Varivax (Oka/Merck), a live attenuated varicella vaccine derived from the vOka strain which later received European approval in 2003. Clinical data confirmed its favorable safety profile and tolerability with minimal vaccine-related adverse events [19,20]. Following this, VarilRix (Oka/GSK) gained FDA approval in 2006, demonstrating high clinical efficacy in pediatric populations during trials [21]. In 2008, BCHT’s live attenuated varicella vaccine was approved in China [22]. Phase III trials of SINOVAC’s live attenuated varicella vaccine achieved a 97.1% seroconversion rate in children aged 1–12 years, with protective efficacy rates of 87.1% against confirmed varicella, 89.2% against breakthrough cases, and 100% against moderate-to-severe disease, while maintaining a favorable safety profile [23]. This vaccine received Chinese approval and attained World Health Organization (WHO) prequalification (PQ) in 2019 [23]. Recent advancements include clinical studies by the Shanghai Institute of Biological Products in 2022, which established strong immunogenicity and safety in individuals ≥13 years using a two-dose regimen with a 4–8 week interval. Consequently, the CDE broadened the vaccine’s target population in 2024 to include all individuals ≥12 months [24].

Beyond the vOka strain, alternative vaccine strains have been developed. The MAV/06 strain, first developed in the 1990s, exhibits significant genetic similarity with the vOka strain. Since 1998, Suduvax^®^ (GC Biopharma), a live attenuated vaccine utilizing the MAV/06 strain, has been commercially available worldwide [25]. More recently, in 2020, BARYCELA^®^ (GC Biopharma), a novel MAV/06-based vaccine, received approval from the Korean Ministry of Food and Drug Safety (KMFDS) [26]. These MAV/06-based vaccines potently induce both humoral and cellular immunity through enhanced Th1-mediated immune responses [26,38]. They elicit robust antibody responses and cross-immunity against circulating Oka strains, demonstrating comparable efficacy to commercial vOka-based vaccines [26,38].

Recent years have witnessed significant advancements in the development of varicella vaccines. In 2022, Wang et al. engineered VZV-7D, a novel live attenuated varicella vaccine with dual attenuation properties targeting both skin and neural tissues [39]. The Phase IIa clinical trial results, published in 2024, revealed that VZV-7D induced humoral immunogenicity comparable to the vOka vaccine in children aged 3–12 years, while maintaining excellent tolerability, underscoring its potential as a safer next-generation live attenuated varicella vaccine [27]. Regulatory progress includes the CDE approval of BOAOVAX’s live attenuated varicella vaccine for Investigational New Drug (IND) status in January 2024 [28]. Furthermore, in December 2024, the CDE granted IND acceptance for Zhejiang Toyouvax Biopharming’s live attenuated varicella vaccine candidate [29].

The live attenuated varicella vaccine is routinely administered subcutaneously. Current vaccination guidelines recommend administering the first dose to children aged 12–18 months [40]. In China, while a single dose demonstrates 75% efficacy in children aged 1–12 years, its efficacy declines significantly in those aged ≥6 years [41]. In the United States, outbreaks continue to occur among highly vaccinated students following single-dose administration, with breakthrough cases occurring 3.4 times more frequently than with two-dose regimens [42]. Therefore, since 2006, the Advisory Committee on Immunization Practices (ACIP) has advocated for a second dose for children aged 4–6 years to establish long-term immunity [42]. Notably, two-dose vaccination achieves 94.9% protective efficacy, representing an approximate 20% increase over single-dose administration [43].

### 2.2. Varicella Combination Vaccines

Advances in varicella combination vaccines have broadened immunization strategies. Typically, the live attenuated varicella vaccine is formulated with the measles, mumps, and rubella (MMR) vaccine to produce a quadrivalent MMRV vaccine. This combined approach simplifies vaccination schedules and enhances coverage rates. Studies have demonstrated that the MMRV vaccine induces immunogenicity comparable to separately administered MMR and varicella (MMR + V) vaccines [44].

The introduction of the MMRV vaccine has significantly influenced public health in the United States. Vaccination coverage rates among children aged 19–35 months have surpassed 90% for either the quadrivalent MMRV or the separate trivalent MMR + V vaccines [30]. Following widespread adoption of the vaccination program, the incidence rates for measles, mumps, rubella, and varicella have decreased substantially, falling to approximately 0.14%, 1.05%, 0.01%, and 10% of pre-vaccine levels, respectively [30].

Since the mid-2000s, two MMRV vaccines have been commercially available: ProQuad^®^ (Merck) and Priorix-Tetra^®^ (GSK) [30]. These formulations differ in their live virus strain compositions. ProQuad incorporates the Edmonston strain (measles), Jeryl Lynn™ strain (mumps), Wistar RA 27/3 strain (rubella), and Oka/Merck strain (varicella), whereas Priorix-Tetra utilizes the Schwarz strain (measles), RIT 4385 strain (a Jeryl Lynn™ derivative for mumps), Wistar RA 27/3 strain (rubella), and Oka/RIT strain (varicella). The recommended MMRV vaccination protocol follows a two-dose schedule: the first dose is administered at 12–15 months to establish immunity before preschool, and the second dose is given at 4–6 years, consistent with preschool vaccination requirements.

A ten-year follow-up study in Russia assessed the efficacy, antibody persistence, and safety of single-dose (Priorix, GSK + Varilrix, GSK) and two-dose (Priorix-Tetra, GSK) vaccination regimens in children. The two-dose regimen achieved 92.4% vaccine efficacy, significantly higher than the 74.7% efficacy observed with single-dose administration [45]. Moreover, anti-VZV seropositivity rates remained above 99.4% in two-dose recipients, compared to 89.7% in single-dose recipients, with comparable safety profiles between groups [45]. These data highlight the clinical benefits of multi-dose vaccination strategies in optimizing immune protection and safety.

## 3. HZ Vaccines

Accumulating clinical evidence underscores the pivotal role of T-cell immunity in preventing primary infection and viral reactivation [46]. Notably, CD4+ T-cell function exhibits a strong association with HZ pathogenesis, while CD8+ T-cells also contribute to viral control during active HZ by targeting VZV-infected cells [47]. Comparative studies reveal that HZ patients display significantly reduced CD4+ T-cells counts and a diminished CD4+/CD8+ T-cell ratio, with severe cases showing more pronounced CD4+ T-cell depletion relative to mild and moderate cases [46]. These findings collectively indicate that eliciting a robust CD4+ T-cell response is essential for enhancing HZ vaccines efficacy, and optimal protection may benefit from coordinated CD4+ and CD8+ T-cell activation.

In recent years, HZ vaccines have emerged as a crucial tool for preventing and managing HZ and its complications. The development of HZ vaccines employs multiple technological platforms, including traditional live attenuated and inactivated vaccines, as well as novel approaches such as subunit, mRNA, viral vector, and virus-like particle (VLP) vaccines (Table 2). These platforms vary significantly in efficacy, safety, immunogenicity, and production efficiency, reflecting their unique mechanisms of action and potential clinical applications. This technological diversity facilitates flexible vaccination strategies for different populations and informs future vaccine optimization and innovation.

### 3.1. Approved HZ Vaccines

#### 3.1.1. Live Attenuated HZ Vaccine (ZVL)

Live attenuated HZ vaccines, such as Zostavax, differ from varicella vaccines in both immunological mechanisms and antigenic dosage, containing significantly higher concentrations of VZV. Specifically, Zostavax is formulated with a minimum of 19,400 PFU (plaque-forming units) per dose, compared to Varivax’s minimum of 1350 PFU [59,60]. This increased viral load is essential to stimulate a robust immune response sufficient to mitigate HZ risk.

Before the introduction of Shingrix (GSK), Zostavax (Merck), a live attenuated HZ vaccine (ZVL), served as the primary vaccine for individuals aged ≥60 years. Levin et al. reported that Zostavax significantly boosted VZV-specific cell-mediated immunity (CMI) and antibody responses in this population, with CMI enhancement lasting up to three years and being more pronounced among individuals aged 60–69 years [61]. However, its protective efficacy diminished progressively with increasing age [62], declining from 63.9% in 60–69-year-olds to 37.6% among those aged ≥70 after a single 0.65 mL subcutaneous injection [15]. Notably, Adriana et al. found that a second dose could enhance and prolong CMI responses [62]. In clinical trials, Zostavax exhibited a 63.6% incidence of injection-site reactions within 5 days post-vaccination [63]. Adverse reactions with an incidence of ≥1% included pain (53.9%), erythema (48.1%), swelling (40.4%), pruritis (11.3%), warmth (3.7%), hematoma (1.6%), and induration (1.1%) [63].

Two additional live attenuated HZ vaccines, SkyZoster (SK Bioscience) and Canvar (BCHT), have received regulatory approval. SkyZoster was first approved by the KMFDS in September 2017, followed by authorization from the Malaysian National Pharmaceutical Regulatory Agency (NPRA) on January 9, 2023. A Phase III trial demonstrated SkyZoster’s clinically acceptable immunogenicity and safety profiles [31], with a 2.75-fold increase in anti-VZV antibody titers observed among 824 vaccinated adults ≥50 years. Furthermore, SkyZoster demonstrated non-inferiority to Zostavax in eliciting CMI responses, while maintaining comparable adverse reaction rates within six weeks post-vaccination and no reported serious vaccine-related events through 26 weeks. Meanwhile, BCHT’s single-dose live attenuated HZ vaccine, Canvar^®^, received market approval on June 3, 2023. Canvar is intended for individuals aged ≥40 years, filling a critical gap in HZ vaccination for the 40–50 age group. Canvar achieved 57.6% overall efficacy against HZ in recipients ≥40 years [32], with age-stratified rates of 37.4% (40–49 years), 62.7% (50–59 years), 64.4% (60–69 years), and 18.6% (≥70 years). The vaccine’s safety profile was favorable, with common adverse reactions (e.g., fever) occurring in <10% of vaccinees [32,64].

While ZVL vaccination is generally considered safe, with most vaccine-associated HZ cases presenting as mild and uncomplicated, breakthrough infections remain possible. Tseng et al. reported the first case of a vaccinated 68-year-old immunocompetent adult with prior wild-type VZV infection who subsequently developed mild, uncomplicated HZ caused by the vaccine strain [65]. In immunocompromised patients, including those who received low-dose immunosuppressive therapy, systemic vOka strain infections may develop, potentially progressing to multi-organ failure [66]. An Australian surveillance study (2016–2020) of 1,089,966 ZVL doses reported 854 adverse events following immunization (AEFI) (78.4/100,000 doses), including 14 disseminated VZV infections (6 confirmed and 8 suspected), yielding a confirmed incidence rate of 0.55/100,000 doses. Notably, 5 of 6 confirmed cases occurred among immunocompromised individuals, with 3 resulting in fatalities. Moreover, studies have identified an association between ZVL vaccination and elevated giant cell arteritis incidence, particularly for clinically and pathologically confirmed cases [67]. These findings highlight the risk of life-threatening vaccine-strain infections in severely immunocompromised individuals, emphasizing the need for pre-vaccination immune evaluation and rigorous post-vaccination surveillance [68].

#### 3.1.2. Recombinant Subunit HZ Vaccine (RZV)

Recombinant zoster vaccine (RZV) induces immunity through recombinant VZV antigenic proteins, thereby eliminating the requirement for intact viral particles. Current RZVs primarily target glycoprotein E, the most abundant VZV envelope protein [69,70]. Functionally, gE forms a heterodimer complex with gI, acting as an Fc receptor in infected cells, which facilitates viral recognition, dissemination, and immune evasion [71]. gE’s close association with host infection mechanisms renders it an optimal antigenic target for HZ vaccine design. Unlike live attenuated vaccines, RZV eliminates the potential for vaccine-strain breakthrough infections or reactivation, while effectively eliciting targeted antibody production and CMI responses.

Shingrix (GSK) is the most widely utilized recombinant zoster vaccine. The FDA granted approval to Shingrix (HZ/Su or GSK1437173A), a two-dose recombinant HZ vaccine, on 20 October 2017 [72]. The vaccination regimen consists of two 0.5 mL intramuscular doses administered 2–6 months apart [73]. This formulation combines recombinant gE with the proprietary AS01B adjuvant system. As the principal target of neutralizing antibodies, gE plays a pivotal role in vaccine efficacy. AS01B are composed of liposomes, monophosphoryl lipid A (MPL) and QS-21 (a saponin derived from Quillaja saponaria), enhancing immunogenicity by stimulating antigen-presenting cells, thereby promoting antibody production and T-cell responses.

On 25 October 2017, ACIP recommended Shingrix for use in immunocompetent adults aged ≥50 years. Although mild local and systemic adverse reactions are common, Shingrix maintains an overall favorable safety and tolerability profile [74]. In clinical studies, Shingrix-induced humoral and CMI responses persisted above pre-vaccination levels for at least 9 years in adults ≥60 years, with modeling data predicting protection lasting up to 15 years [75]. Post-marketing surveillance demonstrated vaccine efficacy rates of 70.1% among ≥65-year-olds receiving the two-dose regimen versus 56.9% for single-dose recipients [76]. Importantly, Shingrix has shown to be effective in immunocompromised populations [76].

The intensity of gE-specific immune responses varies with adjuvant dosage [77]. However, Shingrix’s adjuvant system is associated with side effects, and the scarcity of QS-21 constrains widespread use. Common adverse events comprise pain and fatigue, with 85% of recipients reporting local or systemic reactions in pre-licensure clinical trials [78].

Although rare, individual responses to Shingrix exhibit considerable variability. Schmidt and Maitland reported a 63-year-old male patient who developed acute immune thrombocytopenia (ITP) within two weeks post-vaccination, which subsequently resolved following dexamethasone and intravenous immunoglobulin therapy [79]. This case represents the first reported instance of Shingrix^®^-induced ITP. Despite its well-established efficacy, continued research remains essential to comprehensively characterize its side effects and interindividual response.

RZV is a safe and effective option for immunocompromised patients, offering the potential to reduce the health and socioeconomic burden of HZ [80]. Dagnew et al. reported 87.2% protective efficacy against HZ in immunocompromised patients with hematologic malignancies, with a favorable safety profile [81]. This robust protection highlights RZV’s potential as a preferred alternative to liveattenuated vaccines in immunocompromised populations. Although breakthrough infections following RZV vaccination are exceedingly rare, certain high-risk subgroups may remain vulnerable. For instance, Chen et al. reported the first case of VZV infection manifesting as acute retinal necrosis in an immunocompromised woman after a single Shingrix dose [82]. The cases highlight the importance of tailored vaccination strategies, considering individual treatment regimens to ensure safety and maximize protective efficacy in high-risk populations.

### 3.2. Clinical HZ Vaccines

#### 3.2.1. Recombinant Subunit HZ Vaccine (RZV)

Recent advances in the development of RZV have focused on optimizing immunogenicity and safety profiles beyond the benchmarks set by Shingrix.

Several companies are advancing antigen innovations to boost immune responses. Among these, LZ901, developed by Beijing Luzhu Biotechnology Co., Ltd. and GNW002, developed by Genavax, both utilize gE-Fc fusion proteins as antigens. LZ901 is the first global zoster vaccine featuring a tetrameric molecular structure in an Fc fusion protein format, adjuvanted with aluminum [33]. Extensive preclinical studies and Phase I/II clinical trials in China have demonstrated its high immunogenicity, efficacy, and safety [33]. The FDA granted IND approval for LZ901 in July 2022, and its Phase I trial in the United States commenced in February 2023. Meanwhile, GNW002, a Chinese hamster ovary (CHO) cell-based RZV, employs a dual-adjuvant platform and a gE dimer fusion protein, showing preclinical safety and immune responses comparable to Shingrix [52]. GNW002 received IND approvals in China and the United States in January 2024, with its Phase I trial in China initiating in September 2024. Another candidate, LYB004, a nanoparticle-based zoster vaccine developed by Patronus Biotech using its proprietary U-VLP™ platform, received approval for clinical trials from CDE in July 2024 [28]. Additionally, Gentize Biopharma (Nanjing) developed an RZV using DNA recombination technology to express recombinant gE in CHO cells, demonstrating significant protective efficacy against zoster. Its IND application was accepted by the CDE in September 2024 [28,56].

In addition to advancements in antigen innovation, several companies have developed novel adjuvant systems to enhance immune responses. Maxvax’s CHO cell-based RZV, which incorporates the novel composite adjuvant MA105, demonstrated favorable safety, tolerability, and immunogenicity in Phase I/II clinical trials, with a Phase III trial initiated in July 2024 [35]. Meanwhile, REC610, developed by Recbio and formulated with the novel adjuvant BFA01, showed acceptable safety and tolerability in a Phase I trial in the Philippines involving healthy adults aged ≥40 after two doses [34]. REC610 elicited robust gE-specific humoral and cellular immune responses, detectable immediately after the first dose and peaking 30 days after the second dose, with immune responses comparable to Shingrix [34]. REC610 entered Phase III trials in China in October 2024. Furthermore, Grand Theravac Life Science Nanjing Co. Ltd. developed an RZV incorporating the novel adjuvant system TVA01, which elicited superior cellular and humoral immune responses compared to Shingrix in preclinical studies, alongside favorable safety profiles [28]. This vaccine received approval from the Australian Human Research Ethics Committee in August 2023, leading to a Phase I trial in Australia, followed by a Phase I trial in China in December 2024.

Several additional RZVs are currently under development, though some details remain undisclosed. In June 2024, Dynavax commenced a Phase I/II randomized, observer-blind, active-controlled, dose-escalation, multicenter trial in Australia for its CpG 1018-adjuvanted zoster vaccine (z-1018) to assess safety, tolerability, and immunogenicity compared to Shingrix [48]. Separately, EuBiologics’s EuHZV demonstrated an efficacy rate exceeding 90% in its Phase I trial, which began in the United States in July 2024 [53].

#### 3.2.2. mRNA Vaccines for Herpes Zoster

mRNA vaccines employ nucleic acid fragments encoding VZV antigens, which direct host cells to produce specific antigenic proteins. This mechanism induces robust and durable humoral and cellular immune responses. The distinct antigen expression pattern and generation sites in mRNA vaccines facilitate strong CMI, offering significant potential for HZ vaccines development [83]. Additionally, mRNA vaccines offer enhanced safety profiles by eliminating live viral components, thereby reducing infection risks and substantially accelerating vaccine development and production timelines.

Since 2020, the extensive clinical use of mRNA vaccines developed by Moderna and BioNTech for COVID-19 has demonstrated their safety profile and protective efficacy. This success has validated mRNA technology as a promising vaccine platform, stimulating worldwide research interest in developing mRNA-based vaccines for HZ.

Several investigational candidates are currently undergoing early-stage clinical trials to evaluate their safety, tolerability, and preliminary efficacy. Moderna’s HZ vaccine candidate, mRNA-1468, induced strong antigen-specific T-cell responses one month after a second dose in Phase I/II trials, with favorable tolerability supporting its continued clinical development for HZ prevention [49]. Separately, Pfizer and BioNTech initiated a Phase I/II trial for their mRNA-based HZ vaccine candidate in February 2023 [50,51]. Additionally, Innorna’s mRNA vaccine IN00 1receieved FDA approval for clinical trials in September 2023, followed by clearance from CDE in July 2024, enabling the commencement of its Phase I study [54].

Recent advancements in mRNA vaccine technology have highlighted the unique advantages of self-replicating RNA (srRNA), which enables substantial dose reduction while enhancing antigen expression levels [84]. Immorna’s mRNA-based HZ vaccine candidate, JCXH-105, utilizes the intrinsic adjuvant properties of srRNA to activate innate immune cells, thereby promoting CD4+ T-cell activation and memory cell generation [85]. This immunogenic mechanism may potentially prevent HZ recurrence. Following FDA approval, JCXH-105 entered Phase II trials on 16 October 2024 [86].

Several additional candidates have received IND approvals. In October 2024, Rhegen’s self-developed lyophilized mRNA vaccine for HZ received implicit approval from the CDE, becoming the world’s first lyophilized mRNA vaccine for HZ with independent intellectual property rights [57]. Subsequently, in November 2024, the CDE accepted the IND application for CSPC Pharmaceutical Group Limited’s mRNA vaccine, SYS6017 [28]. On 25 December 2024, the NMPA approved the clinical trial application for SINOVAC’s lyophilized mRNA vaccine against HZ, which is designed to induce immunity against VZV in individuals aged ≥40 [58].

#### 3.2.3. Inactivated HZ Vaccines

Inactivated HZ vaccines are produced by chemically or physically inactivating VZV, preserving its immunogenicity while eliminating its pathogenicity. This process enables the vaccine to safely stimulate an immune response, providing protection against VZV without infection risk. Despite their potential, these vaccines exhibit relatively low immunogenicity. However, incorporating novel adjuvants and advanced delivery systems offers a promising approach to enhance their immune efficacy. To date, no inactivated HZ vaccine has obtained regulatory approval.

Immunocompromised individuals face an increased risk of VZV infection, making inactivated HZ vaccines a safe and practical preventive alternative for this population. Clinical studies have shown that inactivated VZV vaccines (ZVIN) induce strong VZV-specific immune responses in immunocompromised or immunodeficient adults, including those with autoimmune diseases, patients with solid tumors undergoing chemotherapy, and individuals receiving anti-CD20 monoclonal antibody therapy for hematologic malignancies [87,88,89]. These vaccines exhibit favorable safety, tolerability, and efficacy profiles. A pivotal Phase III trial demonstrated that V212 (Merck’s inactivated HZ vaccine) reduced the incidence of HZ by 64% in immunocompromised participants [36]. Further comprehensive clinical trials, combined with long-term studies, are needed to evaluate the clinical effectiveness of inactivated HZ vaccines and assess the influence of multi-dose vaccination strategies on the durability of protective immunity.

#### 3.2.4. Herpes Zoster Viral Vector Vaccine

HZ viral vector vaccine incorporates specific gene fragments of VZV into a viral vector to elicit an immune response. This strategy leverages the vector virus’s natural infectivity for efficient delivery and presentation of VZV antigens (predominantly gE), thereby effectively stimulating the host immune system. The flexibility of viral vectors allows for genetic engineering to optimize immunogenicity and stability, further enhancing their therapeutic potential. Viral vector vaccines are designed to combine the immunogenicity of live attenuated vaccines with the safety profile of subunit vaccines, offering a safer and more effective immunization strategy for diverse populations.

Commonly used viral vectors, such as adenovirus or vaccinia virus, are genetically modified to maximize human safety while maintaining high-efficiency antigen expression and potent immune activation. Preclinical studies of the adenovirus-vectored HZ vaccine candidate CS-2032, co-developed by CanSinoBIO and Vaccitech, demonstrated its ability to induce both humoral and cellular immunity [55]. Notably, the resulting humoral immune response was comparable to that induced by Shingrix, while the cellular immune response was significantly enhanced [55]. A Phase I trial of CS-2032 commenced in Canada in November 2023.

### 3.3. Technical Approaches to Novel HZ Vaccines

Future research on ZVL should focus on exploring novel administration routes to enhance immunogenicity and safety. Studies have shown that intradermal (ID) administration significantly elevates VZV-specific antibody titers compared to subcutaneous (SC) delivery at equivalent doses [90]. Notably, even one-fifth of the intradermal dose can elicit a VZV-specific cellular immunity comparable to or exceeding that achieved via SC injection [91]. Additionally, advancements in vaccine preparation, such as the recombinant vOka bacterial artificial chromosome (rvOka-BAC) system for recombinant live vaccines, offer promising strategies to improve efficacy and safety profiles [92]. Notably, research by Gershon et al. revealed that low levels of constitutive interleukin-10 (IL-10) enhance both humoral immunity and VZV-specific T-cell responses induced by Zostavax, suggesting that pre-vaccination IL-10 modulation could enhance vaccine efficacy [93]. These findings provide valuable insights for optimizing live attenuated HZ vaccines.

Recent advancements in vaccine technology have accelerated the development of RZVs, driven by innovations in immunogen design, adjuvant systems and delivery platforms to enhance immunogenicity. For instance, a novel HZ vaccine incorporating a fusion antigen of glycoprotein E and I (gEgI) adjuvanted with XUA09C demonstrated superior early immune activation, B- and T-cell proliferation and memory T-cell augmentation compared to Shingrix, with stronger cellular immune responses observed in both adult and aged murine models [94]. Similarly, a VZV-gE vaccine formulated with an LNP-CpG ODN adjuvant (a lipid nanoparticle encapsulating VZV-gE and CpG oligodeoxynucleotide) demonstrated comparable immunogenicity and CMI to Shingrix [95]. Notably, all components of this vaccine are scalable for production, highlighting its potential as a cost-effective alternative for HZ prevention. Further optimization of vaccine efficacy relies on precise identification and targeting of immunodominant epitopes. Studies show a truncated gE protein (tgE, aa 31-358) expressed in *Escherichia coli* (*E. coli*) with AS01B adjuvant induced antibody and CMI responses comparable to Shingrix, especially for IFN-γ+ CD4+ T-cells [96]. This *E. coli* system provides a practical gE production method. Among the gE fragments tested, tgE350 showed particularly strong neutralization, informing truncated gE-based vaccine design [97]. Emerging protein engineering and nanoparticle technologies are expected to further improve the potency and stability of next-generation subunit vaccines.

The development of mRNA vaccines presents significant advantages, primarily due to their inherent flexibility, which enables rapid adaptation to genetic variations while enhancing efficacy. Optimizing the gE sequence can further enhance vaccine immunogenicity. Studies indicate that an mRNA-LNP encoding a C-terminal double mutant of gE—engineered to increase viral spread and titer—elicits humoral and cellular immune responses comparable to or stronger than those of Shingrix [98]. Moreover, an LNP-encapsulated mRNA vaccine, gE-M-P, developed by fusing the gE-M nucleic acid sequence with the untranslated region (UTR) of Pfizer/BioNTech’s BNT162b2, has induced robust humoral and cellular immune responses in mice [99]. Further optimization of a full-length gE antigen-encoding VZV mRNA vaccine through signal peptide replacement, C-terminal modification, and mRNA stabilization motifs has substantially enhanced immunogenicity, resulting in superior gE-specific antibody responses, memory B-cell activation and Th1-type T-cell responses across multiple mouse models [100].

In mRNA vaccine development, novel adjuvants and LNP delivery systems offer promising strategies for improving HZ prevention. Research has demonstrated that the adjuvant CIA09, which composed of 1,2-dioleoyl-3-trimethylammonium-propane (DOTAP)-based cationic liposomes combined with the Toll-like receptor 4 agonist de-O-acylated lipooligosaccharide (dLOS), enhances antigen uptake, lymph node delivery, and antigen presentation, thereby strengthening the immune response against VZV gE and promoting a Th1-biased immune response [101]. Similarly, ionizable LNPs amplify the synergistic adjuvant effects of CpG-ODNs and QS21 on VZV gE, improving both humoral and cellular immunity [102]. The HZ vaccine combining VZV gE with the LNP-CpG-QS21 adjuvant system shows potential as a novel alternative to Shingrix, with high cholesterol content in LNPs mitigates the cytotoxicity of QS21 [102]. Both CIA09 and LNP-CpG-QS21 may serve as promising adjuvant candidates for augmenting mRNA vaccine efficacy.

Although HZ viral vector vaccines remain in experimental phases, preliminary data suggest their potential to elicit robust immune protection. A recombinant baculovirus vector vaccine, AcHERV gE-gB, induced total IgG, IgG2a, and neutralizing antibodies levels in mouse models that were comparable to or exceeded those produced by the vOka vaccine, while also stimulating strong VZV-specific CMI [103]. Future research should focus on developing safe and efficient viral vectors, optimizing antigen delivery and immune responses, and assessing their compatibility with existing vaccines.

HZ VLP vaccines, which display conserved VZV epitopes, represent a promising novel immunization strategy. Recombinant hybrid transposon yeast-VLPs (Ty-VLPs) expressing the immunodominant sequence of VZV gE elicit robust humoral and cellular immune responses, demonstrating neutralizing activity against VZV in small animal models [104]. Similarly, another study showed that Ty-VLPs presenting VZV gE or assembly protein fragments act as potent immunogens, stimulating strong immune responses, and were the first to induce specific lymphocyte responses to VZV assembly protein in varicella and HZ patients [85]. Furthermore, Zhu et al. utilized recombinant hepatitis B virus core (HBc)-VLPs to confirm the immunogenicity and neutralizing activity of a highly conserved gE epitope (residues 121–135), providing insights for epitope-based HZ vaccine design [105]. Additionally, a nanoparticle-based HZ vaccine, FR-gE, has also been shown to induce stronger neutralizing antibody responses than both live attenuated vaccines and Shingrix [106]. Future studies should focus on expanding the VLP platform, enhancing immunization strategies, and validating their efficacy to establish VLPs as a viable alternative in HZ vaccine development.

## 4. Discussion

VZV vaccines have significantly advanced the prevention of varicella (chickenpox) and HZ. Their global implementation has led to a substantial reduction in both the incidence and complications associated with these diseases. 

Varicella vaccine development has primarily utilized live attenuated technology, which is now well-established. While this approach has demonstrated good tolerability and immunogenicity in healthy populations, attenuated VZV retains replicative capacity, and may cause breakthrough infections. Future research should investigate VZV virulence factors and attenuation mechanisms, with particularly emphasis on identifying key virulence determinants to enable safer vaccine design. Novel varicella vaccines or sequential immunization strategies should be explored to overcome diminished immune responses and breakthrough infections in immunocompromised individuals. Continued innovation, incorporating emerging technologies, will be crucial for the future development of varicella vaccines.

Similarly, both ZVLs and RZVs have reached advanced development stages. Shingrix has demonstrated high efficacy in high-risk populations and the elderly, with minimal breakthrough infection risk. Current research prioritizes novel vaccine platforms, with key focuses for RZVs including immunogen redesign [33], more potent adjuvant development [34], and alternative administration route exploration [91]. In mRNA vaccine development, efforts concentrate on sequence optimization [37] and LNP-based delivery systems advancement [54]. VLP vaccine efficacy depends on antigen structures optimization [106], while viral vector vaccine progress depends on novel vector identification [55]. These innovative platforms show potential for enhanced safety and durable immune protection, though large-scale clinical trials remain necessary to verify their efficacy and clinical applicability.

Despite its general safety and efficacy, the vOka vaccine strain maintains replicative capacity and can infect host cells, potentially establishing latency in dorsal root ganglia and transmitting to susceptible individuals. The administration of live attenuated varicella vaccines in immunocompromised individuals presents particular challenges due to elevated risks of vaccine-strain infection and transmission, which may result in severe or even fatal complications [107,108]. Documented cases include fatal infections in an immunodeficient child and a stem cell transplant recipient with non-Hodgkin lymphoma [109,110]. Among immunocompromised patients, including those receiving low-dose immunosuppressive therapy, ZVL vaccination may cause systemic infection by the vaccine strain of VZV, potentially leading to multi-organ failure [66]. These cases highlight the need for rigorous risk assessment and careful clinical evaluation when considering live attenuated vaccines for immunocompromised populations. Although live attenuated VZV vaccines carry inherent risks, their demonstrated protective benefits and manageable safety profile support their continued inclusion in routine immunization programs.

Unlike ZVL, breakthrough infections associated with RZV are exceedingly rare. RZV represents a safe and effective option for immunocompromised individuals, with potential to mitigate the health and socioeconomic burdens of HZ [80]. Studies have shown that RZV is well-tolerated, safe, and effective in immunocompromised populations such as autologous hematopoietic stem cell transplant recipients and patients with hematologic malignancies [81,111]. Furthermore, emerging next-generation vaccine technologies, particularly mRNA-based platforms, may offer improved safety and immunogenicity in future vaccine development.

The COVID-19 pandemic has introduced new complexities in vaccine management and efficacy, with reports of VZV reactivation following COVID-19 vaccination [112,113,114]. These observations highlight the need for further investigation into vaccine interactions and their immunological consequences. Anti-SARS-CoV-2 vaccines may modulate immune responses, potentially influencing VZV reactivation, particularly in individuals with age-related immune decline (immunosenescence). Consequently, vaccination strategies should account for these immunological considerations. Pre-vaccination immune assessments, particularly in high-risk populations, as well as post-vaccination surveillance, are essential to mitigate adverse effects and transmission risks.

Furthermore, the efficacy of these vaccines may be influenced by recipients’ baseline immune status before vaccination. Obesity has been linked to reduced seroconversion rates, potentially impairing vaccine-induced immune responses [115]. Likewise, untreated depression may increase the risk and severity of HZ while also reducing vaccine effectiveness. In contrast, antidepressant therapy can restore immune function and potentially improve vaccine efficacy [116]. These findings emphasize the critical role of maintaining overall health to support optimal antiviral immunity.

The success of VZV vaccines underscores their potential as platforms for broader infectious disease prevention. Given its ability to express foreign genes, VZV serves as a versatile viral vector in vaccine design, offering potential for a wide range of viral infections. VZV-based vaccines have been explored in HIV vaccine development, demonstrating potential to enhance both systemic and genital mucosal immunity [117]. Similarly, a VZV vector expressing simian immunodeficiency virus (SIV) antigens has demonstrated a 37.5% protection rate against pathogenic SIV challenges by eliciting mucosal memory, humoral, and cellular immune responses [118]. These findings underscore the potential of VZV as a robust platform for developing vaccines against immunodeficiency viruses. Furthermore, a recombinant VZV expressing Epstein–Barr virus (EBV) membrane glycoprotein (gp350/220) has emerged as a promising vector for active immunization against EBV and other pathogens [119].

In summary, vaccination against varicella and HZ remains a critical public health strategy, offering substantial benefits in preventing severe disease and reducing mortality. The benefits of VZV vaccination significantly outweigh the minimal risks of adverse effects. However, current research identifies key gaps in VZV vaccine development, particularly in enhancing vaccine diversity, efficacy, and safety. The continued development and refinement of safe and effective VZV vaccines, along with increased vaccination coverage, can substantially reduce varicella and HZ incidence, prevent severe complications, establish herd immunity, interrupt viral transmission, and mitigate healthcare costs. Continued scientific advancements and technological innovations show considerable promise for enhancing the public health benefits of VZV vaccines, ultimately strengthening global immunization efforts and contributing to long-term disease control.

## Figures and Tables

**Table 1:** Approved and clinical varicella vaccines: monovalent and combination formulations.

Vaccine Type	Development Progress	Manufacturer/Country	Vaccine Name	Main Vaccine Components
Monovalent vaccine	Approved	Biken/Japan [18]	OkaVax	Oka strain
		Merck/Germany [19,20]	Varivax	Oka strain
		GlaxoSmithKline (GSK)/UK [21]	Varilrix	Oka strain
		BCHT/China [22]	\	Oka strain
		SINOVAC/China [23]	\	Oka strain
		Shanghai Institute of Biological Products Co., Ltd./China [24]	\	Oka strain
		GC Biopharma/Republic of Korea [25]	Suduvax	MAV/06 strain
		GC Biopharma/Republic of Korea [26]	BARYCELA	MAV/06 strain
	Phase II	Xiamen University (China)/Rutgers New Jersey Medical School (US) [27]	VZV-7D	Oka strain
	IND	BOAOVAX/China [28]	\	Oka strain
Monovalent vaccine	IND	Zhejiang Toyouvax Biopharming/China [29]	\	Oka strain
Combination vaccine	Approved	Merck/Germany [30]	ProQuad	Edmonston strain (measles) Jeryl Lynn™ strain (mumps) Wistar RA 27/3 strain (rubella) Oka/Merck strain (varicella)
		GlaxoSmithKline (GSK)/UK [30]	Priorix-Tetra	Schwarz strain (measles) RIT 4385 strain (a Jeryl Lynn™ derivative for mumps) Wistar RA 27/3 strain (rubella) Oka/RIT strain (varicella)

**Table 2:** Comparison of approved and clinical HZ vaccines by different technological platforms.

Development Progress	Manufacturer/Country	Vaccine Name	Technical Approach	Main Vaccine Components	Adjuvant
Approved	Merck/Germany	Zostavax	ZVL	Oka strain	\
	SK Bioscience/Republic of Korea [31]	SkyZoster	ZVL	Oka strain	\
	BCHT/China [32]	Canvar	ZVL	Oka strain	\
	GlaxoSmithKline (GSK) /UK	Shingrix	RZV	gE/CHO	AS01B
Phase III	Beijing Luzhu Biotechnology Co., Ltd./China [33]	LZ901	RZV	gE-Fc fusion protein/CHO	Alum
	Recbio/China [34]	REC610	RZV	gE/CHO	BFA01
	Maxvax/China [35]	\	RZV	gE/CHO	combined adjuvant MA105
Phase III	Merck/Germany [36]	V212	inactivated vaccine	Inactivated VZV	
Phase II	Immorna/China [37]	JCXH-105	mRNA vaccine	srRNA	the adjuvant effect of srRNA itself
Phase I/II	Dynavax/US [48]	z-1018	RZV	gE/CHO	CpG 1018
	Moderna/US [49]	mRNA-1468	mRNA vaccine	gE mRNA-LNP	LNP
	Pfizer (US)/BioNTech (Germany) [50,51]	VZV modRNA	mRNA vaccine	gE mRNA-LNP	LNP
Phase I	Genevax/China [52]	GNW002	RZV	gE-Fc fusion protein/CHO	Alum+CpG
	Patronus Biotech/China [28]	LYB004	RZV	gE-VLP/CHO	A01
	Grand Theravac Life Science Nanjing Co. Ltd./China [28]	\	RZV	gE/CHO	TVA01
	EuBiologics/Republic of Korea [53]	EuHZV	RZV	gE	EcML+CoPoP
	Innorna/China [54]	IN001	mRNA vaccine	gE mRNA-LNP	LNP
	CanSinoBIO (China)/Vaccitech (UK) [55]	CS-2032	adenovirus vector vaccine	ChOx1 replication-deficient chimpanzee adenovirus vector encoding VZV-gE	
IND	Gentize Biopharma (Nanjing)/China [28,56]	\	RZV	gE/CHO	
	Rhegen/China [57]	Freeze-dried shingles mRNA vaccine	mRNA vaccine	gE mRNA-LNP	LNP
	CSPC Pharmaceutical Group Limited/China [28]	SYS6017	mRNA vaccine	gE mRNA-LNP	LNP
	SINOVAC/China [58]	Freeze-dried shingles mRNA vaccine	mRNA vaccine	gE mRNA-LNP	LNP

ZVL: live attenuated HZ vaccine; RZV: recombinant subunit HZ vaccine.

## Data Availability

All relevant data are within the manuscript.

## Abbreviations

ACIP	Advisory Committee on Immunization Practices
AEFI	Adverse events following immunization
bp	Base pair
CDE	Center for Drug Evaluation
CHO	Chinese hamster ovary (cells)
CMI	Cell-mediated immunity
CpG	Cytosine-phosphate-Guanine
dLOS	de-O-acylated lipooligosaccharide
DOTAP	1,2-dioleoyl-3-trimethylammonium-propane
*E. coli*	*Escherichia coli*
EBV	Epstein–Barr virus
FDA	Food and Drug Administration
gE	Glycoprotein E
gEgI	Glycoprotein E and I
GPE	Guinea pig embryo
HBc	Hepatitis B virus core
HEL	Human embryonic lung
HHV-3	Human herpesvirus 3
HIV	Human immunodeficiency virus
HZ	Herpes zoster
ID	Intradermal
IL-10	Interleukin-10
IND	Investigational New Drug
ITP	Immune thrombocytopenia
KMFDS	Korean Ministry of Food and Drug Safety
LNP	Lipid nanoparticle
LNP-CpG-ODN	Lipid nanoparticle encapsulating CpG-ODN
MMR	Measles–mumps–rubella
MMR + V	Measles–mumps–rubella and varicella
MMRV	Measles–mumps–rubella–varicella
MPL	Monophosphoryl lipid A
NMPA	National Medical Products Administration
NPRA	National Pharmaceutical Regulatory Agency
ODN	Oligodeoxynucleotide
ORF	Open reading frame
PFU	Plaque-forming units
PHN	Postherpetic neuralgia
pOka	Parental Oka strain
PQ	Prequalification
QS-21	A saponin derived from *Quillaja saponaria*
rvOka-BAC	Recombinant vOka bacterial artificial chromosome
RZV	Recombinant zoster vaccine
SC	Subcutaneous
SIV	Simian immunodeficiency virus
srRNA	Self-replicating RNA
tgE	Truncated gE protein
Ty-VLPs	Hybrid transposon yeast-VLPs
UK	United Kingdom
US	United States
UTR	Untranslated region
U-VLP™	Patronus Biotech’s proprietary nanoparticle platform
VLP	Virus-like particle
vOka	Vaccine Oka strain
VZV	Varicella-zoster virus
WHO	World Health Organization
WI-38	Human diploid cell line
ZVIN	Inactivated VZV vaccine
ZVL	Live attenuated HZ vaccine

## References

[1] Davison A.J., Scott J.E. (1986). The complete DNA sequence of varicella-zoster virus. J. Gen. Virol..

[2] Storlie J., Maresova L., Jackson W., Grose C. (2008). Comparative analyses of the 9 glycoprotein genes found in wild-type and vaccine strains of varicella-zoster virus. J. Infect. Dis..

[3] de Martino Mota A., Carvalho-Costa F.A. (2016). Varicella zoster virus related deaths and hospitalizations before the introduction of universal vaccination with the tetraviral vaccine. J. Pediatr..

[4] Lachiewicz A.M., Srinivas M.L. (2019). Varicella-zoster virus post-exposure management and prophylaxis: A review. Prev. Med. Rep..

[5] Galil K., Choo P.W., Donahue J.G., Platt R. (1997). The sequelae of herpes zoster. Arch. Intern. Med..

[6] Schmidt M., Kress M., Heinemann S., Fickenscher H. (2003). Varicella-zoster virus isolates, but not the vaccine strain OKA, induce sensitivity to alpha-1 and beta-1 adrenergic stimulation of sensory neurones in culture. J. Med. Virol..

[7] Sadaoka T., Mori Y. (2018). Vaccine Development for Varicella-Zoster Virus. Adv. Exp. Med. Biol..

[8] Pepose J.S. (1997). The potential impact of the varicella vaccine and new antivirals on ocular disease related to varicella-zoster virus. Am. J. Ophthalmol..

[9] Tirat W.R., Schibler M. (2022). Varicella-zoster virus (VZV) acute retinal necrosis and recombinant zoster vaccine. Rev. Med. Suisse.

[10] Newman A.M., Jhaveri R. (2019). Myths and Misconceptions: Varicella-Zoster Virus Exposure, Infection Risks, Complications, and Treatments. Clin. Ther..

[11] Feigin. R.D., Cherry. J.D., Demmler-Harrison. G.J., Kaplan. S.L. (2009). Feigin and Cherry’s Textbook of Pediatric Infectious Diseases.

[12] Bialek S.R., Perella D., Zhang J., Mascola L., Viner K., Jackson C., Lopez A.S., Watson B., Civen R. (2013). Impact of a routine two-dose varicella vaccination program on varicella epidemiology. Pediatrics.

[13] Shapiro E.D., Vazquez M., Esposito D., Holabird N., Steinberg S.P., Dziura J., LaRussa P.S., Gershon A.A. (2011). Effectiveness of 2 doses of varicella vaccine in children. J. Infect. Dis..

[14] Sheel M., Beard F., Quinn H., Dey A., Kirk M., Koehler A., Markey P., McIntyre P., Macartney K. (2018). Australian vaccine preventable disease epidemiological review series: Varicella-zoster virus infections, 1998-2015. Commun. Dis. Intell..

[15] Bharucha T., Ming D., Breuer J. (2017). A critical appraisal of ‘Shingrix’, a novel herpes zoster subunit vaccine (HZ/Su or GSK1437173A) for varicella zoster virus. Hum. Vaccin. Immunother..

[16] Laing K.J., Ouwendijk W.J.D., Koelle D.M., Verjans G. (2018). Immunobiology of Varicella-Zoster Virus Infection. J. Infect. Dis..

[17] Haberthur K., Engelmann F., Park B., Barron A., Legasse A., Dewane J., Fischer M., Kerns A., Brown M., Messaoudi I. (2011). CD4 T cell immunity is critical for the control of simian varicella virus infection in a nonhuman primate model of VZV infection. PLoS Pathog..

[18] Takahashi M. (2001). 25 years’ experience with the Biken Oka strain varicella vaccine: A clinical overview. Paediatr. Drugs.

[19] Goulleret N., Mauvisseau E., Essevaz-Roulet M., Quinlivan M., Breuer J. (2010). Safety profile of live varicella virus vaccine (Oka/Merck): Five-year results of the European Varicella Zoster Virus Identification Program (EU VZVIP). Vaccine.

[20] Paradis E.M., Tikhonov O., Cao X., Kharit S.M., Fokin A., Platt H.L., Wittke F., Jotterand V. (2021). Phase 3, open-label, Russian, multicenter, single-arm trial to evaluate the immunogenicity of varicella vaccine (VARIVAX™) in healthy infants, children, and adolescents. Hum. Vaccines Immunother..

[21] Sheffer R., Segal D., Rahamani S., Dalal I., Linhart Y., Stein M., Shohat T., Somekh E. (2005). Effectiveness of the Oka/GSK attenuated varicella vaccine for the prevention of chickenpox in clinical practice in Israel. Pediatr. Infect. Dis. J..

[22] BCHT Live Attenuated Vaccine Against Varicella. http://www.bchtpharm.com/Home/Article/detail/id/804.html.

[23] SINOVAC SINOVAC Varicella Attenuated Live Vaccine Has Been Pre Certified by the World Health Organization. http://www.sinovac.com.cn/zh-cn/news/id-3194.

[24] SINOPHARM The Vaccination Age for the Varicella Attenuated Live Vaccine of Shanghai Institute of Biological Products Co., Ltd. Has Been Extended to the Entire Population Aged 12 Months and Above. https://www.sinopharm.com/2024-08/27/c_19710.htm.

[25] Kim J.I., Jung G.S., Kim Y.Y., Ji G.Y., Kim H.S., Wang W.D., Park H.S., Park S.Y., Kim G.H., Kwon S.N. (2011). Sequencing and characterization of Varicella-zoster virus vaccine strain SuduVax. Virol. J..

[26] Shin D., Shin Y., Kim E., Nam H., Nan H., Lee J. (2022). Immunological characteristics of MAV/06 strain of varicella-zoster virus vaccine in an animal model. BMC Immunol..

[27] Pan H.X., Qiu L.X., Liang Q., Chen Z., Zhang M.L., Liu S., Zhong G.H., Zhu K.X., Liao M.J., Hu J.L. (2024). Immunogenicity and safety of an ORF7-deficient skin-attenuated and neuro-attenuated live vaccine for varicella: A randomised, double-blind, controlled, phase 2a trial. Lancet Infect. Dis..

[28] Center For Drug Evaluation, NMPA Disclosure of Recipient Variety Information. https://www.cde.org.cn/main/xxgk/listpage/9f9c74c73e0f8f56a8bfbc646055026d.

[29] Pharmacy B. Announcement of Shandong Stepwise Pharmaceutical Co., Ltd. on the Acceptance Notice of Clinical Trials of Its Controlled Subsidiary’s Drugs. https://www.cnpharm.com/upload/resources/file/2024/12/19/167923.pdf.

[30] Kowalzik F., Faber J., Knuf M. (2018). MMR and MMRV vaccines. Vaccine.

[31] Newswire P. SK Bioscience’s Zoster Vaccine Receives Biologics License Application Approval in Malaysia. https://en.prnasia.com/releases/global/sk-bioscience-s-zoster-vaccine-receives-biologics-license-application-approval-in-malaysia-389846.shtml.

[32] BCHT Live Attenuated Herpes Zoster Vaccine: Canvar^®^. http://www.bchtpharm.com/Home/Article/detail/id/905.html.

[33] Biotech L. Recombinant Herpes Zoster Vaccine (LZ901). http://www.luzhubiotech.com/techAndProduct/productInResearch.

[34] Zhang Z., Liu X., Suo L., Zhao D., Pan J., Lu L. (2023). The incidence of herpes zoster in China: A meta-analysis and evidence quality assessment. Hum. Vaccin. Immunother..

[35] Maxvax Milestones|Phase III Clinical Enrollment of Maikokang Recombinant Herpes Zoster Vaccine (CHO Cells). http://www.maxvax.cn/info.aspx?id=119&t=9.

[36] Merck In First Phase 3 Trial, Merck’s Investigational Inactivated Varicella Zoster Virus Vaccine (V212) Reduced the Incidence of Confirmed Herpes Zoster Cases by an Estimated 64 Percent in Immunocompromised Subjects. https://www.merck.com/news/in-first-phase-3-trial-mercks-investigational-inactivated-varicella-zoster-virus-vaccine-v212-reduced-the-incidence-of-confirmed-herpes-zoster-cases-by-an-estimated-64-percent-in-immunocom/.

[37] Immorna JCXH-105, the World’s First RNA Shingles Vaccine, Has Been Approved for Phase I Registration Clinical Trials by the US Food and Drug Administration. https://www.immorna.com/cn/investors-detail-192.html.

[38] Choi U.Y., Huh D.H., Kim J.H., Kang J.H. (2016). Seropositivity of Varicella zoster virus in vaccinated Korean children and MAV vaccine group. Hum. Vaccin. Immunother..

[39] Wang W., Pan D., Fu W., Ye X., Han J., Yang L., Jia J., Liu J., Zhu R., Zhang Y. (2022). Development of a skin- and neuro-attenuated live vaccine for varicella. Nat. Commun..

[40] Kudesia G., Partridge S., Farrington C.P., Soltanpoor N. (2002). Changes in age related seroprevalence of antibody to varicella zoster virus: Impact on vaccine strategy. J. Clin. Pathol..

[41] Zhang Z.J.Z., Suo L.D., Zhao D., Pan J.B., Lu L. (2020). Systematic reviews and evidence quality assessment on effectiveness of 1 dose varicella attenuated live vaccine for healthy children aged 1-12 years in China. Zhonghua Liu Xing Bing Xue Za Zhi.

[42] Marin M., Güris D., Chaves S.S., Schmid S., Seward J.F. (2007). Prevention of varicella: Recommendations of the Advisory Committee on Immunization Practices (ACIP). MMWR Recomm. Rep..

[43] Prymula R., Bergsaker M.R., Esposito S., Gothefors L., Man S., Snegova N., Štefkovičova M., Usonis V., Wysocki J., Douha M. (2014). Protection against varicella with two doses of combined measles-mumps-rubella-varicella vaccine versus one dose of monovalent varicella vaccine: A multicentre, observer-blind, randomised, controlled trial. Lancet.

[44] Leung J.H., Hirai H.W., Tsoi K.K. (2015). Immunogenicity and reactogenicity of tetravalent vaccine for measles, mumps, rubella and varicella (MMRV) in healthy children: A meta-analysis of randomized controlled trials. Expert Rev. Vaccines.

[45] Namazova-Baranova L., Habib M.A., Povey M., Efendieva K., Fedorova O., Fedoseenko M., Ivleva T., Kovshirina Y., Levina J., Lyamin A. (2022). A randomized trial assessing the efficacy, immunogenicity, and safety of vaccination with live attenuated varicella zoster virus-containing vaccines: Ten-year follow-up in Russian children. Hum. Vaccin. Immunother..

[46] Xing Q., Hu D., Shi F., Chen F. (2013). Role of regulatory T cells in patients with acute herpes zoster and relationship to postherpetic neuralgia. Arch. Dermatol. Res..

[47] Steain M., Sutherland J.P., Rodriguez M., Cunningham A.L., Slobedman B., Abendroth A. (2014). Analysis of T cell responses during active varicella-zoster virus reactivation in human ganglia. J. Virol..

[48] Dynavax Advancing a Broad Pipeline of Vaccines to Prevent Infectious Diseases. https://www.dynavax.com/pipeline/#trials.

[49] Moderna Moderna Advances Multiple Vaccine Programs to Late-Stage Clinical Trials. https://news.modernatx.com/news/news-details/2024/Moderna-Advances-Multiple-Vaccine-Programs-to-Late-Stage-Clinical-Trials/default.aspx.

[50] Pfizer Pfizer and BioNTech Initiate Phase 1/2 Study of First mRNA-Based Shingles Vaccine Program. https://www.pfizer.com/news/announcements/pfizer-and-biontech-initiate-phase-12-study-first-mrna-based-shingles-vaccine.

[51] National Library of Medicine Study Details|A Study to Learn About a Modified RNA Vaccine Against Shingles in Healthy Adults|ClinicalTrials.Gov. https://clinicaltrials.gov/study/NCT05703607.

[52] Genevax Good News: Geneve Recombinant Herpes Zoster Vaccine Has Obtained FDA Clinical Trial Approval!. https://www.genevax.com.cn/newsinfo/6818959.html.

[53] ClinicalTrials.Gov Clinical Trial to Evaluate EuHZV in Healthy Adults Aged 50 to 69 Years. https://clinicaltrials.gov/study/NCT06409494?term=EuHZV&rank=1.

[54] Innorna Innorna Shingles mRNA Vaccine IN001 Has Been Approved for Clinical Trials in China. https://www.innorna.com/cn/news/314.html.

[55] CanSinoBio CanSinoBio Recombinant Herpes Zoster Vaccine Launches Phase I Clinical Trial in Canada and Completes First Subject Enrollment. https://www.cansinotech.com.cn/detail-4188.

[56] Biopharma G. Recombinant Herpes Zoster Vaccine. http://www.gentize.com/product/9.html.

[57] RHEGEN The Clinical Trial Application for the World’s First Freeze-Dried Herpes Zoster mRNA Vaccine Has Been Approved by CDE. https://www.rhegen.com/show-8-33-1.html.

[58] SINOVAC SINOVAC Freeze-Dried Herpes Zoster Virus mRNA Vaccine Approved for Clinical Use. https://www.sinovac.com/zh-cn/news/id-3385.

[59] Food and Drug Administration ZOSTAVAX (Zoster Vaccine Live) Frozen Package Insert. https://www.fda.gov/media/119879/download?attachment.

[60] Food and Drug Administration Clinical Review Memo—VARIVAX. https://www.fda.gov/media/166083/download.

[61] Levin M.J., Oxman M.N., Zhang J.H., Johnson G.R., Stanley H., Hayward A.R., Caulfield M.J., Irwin M.R., Smith J.G., Clair J. (2008). Varicella-zoster virus-specific immune responses in elderly recipients of a herpes zoster vaccine. J. Infect. Dis..

[62] Weinberg A., Popmihajlov Z., Schmader K.E., Johnson M.J., Caldas Y., Salazar A.T., Canniff J., McCarson B.J., Martin J., Pang L. (2019). Persistence of Varicella-Zoster Virus Cell-Mediated Immunity After the Administration of a Second Dose of Live Herpes Zoster Vaccine. J. Infect. Dis..

[63] RxList Zostavax. https://www.rxlist.com/zostavax-drug.htm#side_effects.

[64] DXY Canvar Product Manual. https://drugs.dxy.cn/pc/drug/eu8Irmepepm9Rhzuexg72hUofEA==?ky=%E5%B8%A6%E7%8A%B6%E7%96%B1%E7%96%B9%E7%96%AB%E8%8B%97.

[65] Tseng H.F., Schmid D.S., Harpaz R., LaRussa P., Jensen N.J., Rivailler P., Radford K., Folster J., Jacobsen S.J. (2014). Herpes zoster caused by vaccine-strain varicella zoster virus in an immunocompetent recipient of zoster vaccine. Clin. Infect. Dis..

[66] Dubey V., MacFadden D. (2019). Disseminated varicella zoster virus infection after vaccination with a live attenuated vaccine. CMAJ.

[67] Agger W.A., Deviley J.A., Borgert A.J., Rasmussen C.M. (2021). Increased Incidence of Giant Cell Arteritis After Introduction of a Live Varicella Zoster Virus Vaccine. Open Forum. Infect. Dis..

[68] Li-Kim-Moy J., Phillips A., Morgan A., Glover C., Jayasinghe S., Hull B.P., Dey A., Beard F.H., Hickie M., Macartney K. (2023). Disseminated varicella zoster virus infection following live attenuated herpes zoster vaccine: Descriptive analysis of reports to Australia’s spontaneous vaccine pharmacovigilance system, 2016–2020. BMJ Open.

[69] Cole N.L., Grose C. (2003). Membrane fusion mediated by herpesvirus glycoproteins: The paradigm of varicella-zoster virus. Rev. Med. Virol..

[70] Yao Z., Jackson W., Forghani B., Grose C. (1993). Varicella-zoster virus glycoprotein gpI/gpIV receptor: Expression, complex formation, and antigenicity within the vaccinia virus-T7 RNA polymerase transfection system. J. Virol..

[71] Yao Z., Grose C. (1994). Unusual phosphorylation sequence in the gpIV (gI) component of the varicella-zoster virus gpI-gpIV glycoprotein complex (VZV gE-gI complex). J. Virol..

[72] Dooling K.L., Guo A., Patel M., Lee G.M., Moore K., Belongia E.A., Harpaz R. (2018). Recommendations of the Advisory Committee on Immunization Practices for Use of Herpes Zoster Vaccines. MMWR Morb. Mortal. Wkly. Rep..

[73] Food and Drug Administration Shingrix [Package Insert]. https://www.fda.gov/media/108597/download.

[74] Lal H., Zahaf T., Heineman T.C. (2013). Safety and immunogenicity of an AS01-adjuvanted varicella zoster virus subunit candidate vaccine (HZ/su): A phase-I, open-label study in Japanese adults. Hum. Vaccin. Immunother..

[75] Schwarz T.F., Volpe S., Catteau G., Chlibek R., David M.P., Richardus J.H., Lal H., Oostvogels L., Pauksens K., Ravault S. (2018). Persistence of immune response to an adjuvanted varicella-zoster virus subunit vaccine for up to year nine in older adults. Hum. Vaccin. Immunother..

[76] Izurieta H.S., Wu X., Forshee R., Lu Y., Sung H.M., Agger P.E., Chillarige Y., Link-Gelles R., Lufkin B., Wernecke M. (2021). Recombinant Zoster Vaccine (Shingrix): Real-World Effectiveness in the First 2 Years Post-Licensure. Clin. Infect. Dis..

[77] Chlibek R., Bayas J.M., Collins H., de la Pinta M.L., Ledent E., Mols J.F., Heineman T.C. (2013). Safety and immunogenicity of an AS01-adjuvanted varicella-zoster virus subunit candidate vaccine against herpes zoster in adults >=50 years of age. J. Infect. Dis..

[78] Hesse E.M., Shimabukuro T.T., Su J.R., Hibbs B.F., Dooling K.L., Goud R., Lewis P., Ng C.S., Cano M.V. (2019). Postlicensure Safety Surveillance of Recombinant Zoster Vaccine (Shingrix)—United States, October 2017–June 2018. MMWR Morb. Mortal. Wkly. Rep..

[79] Schmidt N., Maitland H. (2021). Acute Immune Thrombocytopenia following administration of Shingrix recombinant zoster vaccine. Am. J. Hematol..

[80] Ishihara R., Watanabe R., Shiomi M., Katsushima M., Fukumoto K., Yamada S., Okano T., Hashimoto M. (2024). Exploring the Link between Varicella-Zoster Virus, Autoimmune Diseases, and the Role of Recombinant Zoster Vaccine. Biomolecules.

[81] Ljungman P. (2019). Varicella zoster virus vaccine in patients with haematological malignancies. Lancet Infect. Dis..

[82] Chen R.I., Deaner J.D., Srivastava S.K., Lowder C.Y. (2020). Acute retinal necrosis following recombinant subunit varicella-zoster virus vaccine. Am. J. Ophthalmol. Case Rep..

[83] Chaudhary N., Weissman D., Whitehead K.A. (2021). mRNA vaccines for infectious diseases: Principles, delivery and clinical translation. Nat. Rev. Drug Discov..

[84] Vogel A.B., Lambert L., Kinnear E., Busse D., Erbar S., Reuter K.C., Wicke L., Perkovic M., Beissert T., Haas H. (2018). Self-Amplifying RNA Vaccines Give Equivalent Protection against Influenza to mRNA Vaccines but at Much Lower Doses. Mol. Ther..

[85] Welsh M.D., Harper D.R., Garcia-Valcarcel M., Fowler W.J., Aitken C., Jeffries D.J., Layton G.T. (1999). Ability of yeast Ty-VLPs (virus-like particles) containing varicella-zoster virus (VZV)gE and assembly protein fragments to induce in vitro proliferation of human lymphocytes from VZV immune patients. J. Med. Virol..

[86] ClinicalTrials.Gov A Phase 2 Study to Evaluate JCXH-105, an srRNA-Based Herpes Zoster Vaccine. https://clinicaltrials.gov/study/NCT06581575?cond=JCXH-105&rank=1.

[87] Eberhardson M., Hall S., Papp K.A., Sterling T.M., Stek J.E., Pang L., Zhao Y., Parrino J., Popmihajlov Z. (2017). Safety and Immunogenicity of Inactivated Varicella-Zoster Virus Vaccine in Adults With Autoimmune Disease: A Phase 2, Randomized, Double-Blind, Placebo-Controlled Clinical Trial. Clin. Infect. Dis..

[88] Parrino J., McNeil S.A., Lawrence S.J., Kimby E., Pagnoni M.F., Stek J.E., Zhao Y., Chan I.S., Kaplan S.S. (2017). Safety and immunogenicity of inactivated varicella-zoster virus vaccine in adults with hematologic malignancies receiving treatment with anti-CD20 monoclonal antibodies. Vaccine.

[89] Mullane K.M., Morrison V.A., Camacho L.H., Arvin A., McNeil S.A., Durrand J., Campbell B., Su S.C., Chan I.S.F., Parrino J. (2019). Safety and efficacy of inactivated varicella zoster virus vaccine in immunocompromised patients with malignancies: A two-arm, randomised, double-blind, phase 3 trial. Lancet Infect. Dis..

[90] Beals C.R., Railkar R.A., Schaeffer A.K., Levin Y., Kochba E., Meyer B.K., Evans R.K., Sheldon E.A., Lasseter K., Lang N. (2016). Immune response and reactogenicity of intradermal administration versus subcutaneous administration of varicella-zoster virus vaccine: An exploratory, randomised, partly blinded trial. Lancet Infect. Dis..

[91] Nakamura-Nishimura Y., Shinkuma S., Miyagawa F., Haredy A., Gomi Y., Yamanishi K., Asada H. (2022). Immunogenicity of varicella-zoster virus vaccine by different routes of administration: Comparable vaccination efficacy of one-fifth dose intradermal vaccination to conventional subcutaneous vaccination. J. Dermatol. Sci..

[92] Yoshii H., Somboonthum P., Takahashi M., Yamanishi K., Mori Y. (2007). Cloning of full length genome of varicella-zoster virus vaccine strain into a bacterial artificial chromosome and reconstitution of infectious virus. Vaccine.

[93] Gershon A.A., Brooks D., Stevenson D.D., Chin W.K., Oldstone M.B.A., Gershon M.D. (2019). High Constitutive Interleukin 10 Level Interferes With the Immune Response to Varicella-Zoster Virus in Elderly Recipients of Live Attenuated Zoster Vaccine. J. Infect. Dis..

[94] Zhang S., Zeng Y., Cui L., Zhang Y., Chen T., Xue W., Wang H., Liu H., Zhang Y., Chen L. (2025). Immunogenicity and cellular response of a herpes zoster virus gEgI fusion protein adjuvanted with CpG-emulsion in mice. J. Nanobiotechnol..

[95] Luan N., Cao H., Wang Y., Lin K., Liu C. (2022). LNP-CpG ODN-adjuvanted varicella-zoster virus glycoprotein E induced comparable levels of immunity with Shingrix™ in VZV-primed mice. Virol. Sin..

[96] Chen T., Sun J., Zhang S., Li T., Liu L., Xue W., Zhou L., Liang S., Yu Z., Zheng Q. (2023). Truncated glycoprotein E of varicella-zoster virus is an ideal immunogen for Escherichia coli-based vaccine design. Sci. China Life Sci..

[97] Wu J., Li H., Yuan Y., Wang R., Shi T., Li Z., Cui Q., Fu S., Nie K., Li F. (2024). Truncated VZV gE Induces High-Titer Neutralizing Antibodies in Mice. Vaccines.

[98] Cao H., Wang Y., Luan N., Lin K., Liu C. (2021). Effects of Varicella-Zoster Virus Glycoprotein E Carboxyl-Terminal Mutation on mRNA Vaccine Efficacy. Vaccines.

[99] Wang Y., Cao H., Lin K., Hu J., Luan N., Liu C. (2023). Evaluation of the Immunological Efficacy of an LNP-mRNA Vaccine Prepared from Varicella Zoster Virus Glycoprotein gE with a Double-Mutated Carboxyl Terminus in Different Untranslated Regions in Mice. Vaccines.

[100] Huang L., Zhang S., Zhao T., Cai T., Bu L., Di Z., Zhang Y., Yang C., Yang Y., Lin A. (2024). Rational optimization of glycoprotein E (gE)-encoding mRNA for improved Varicella-zoster virus mRNA vaccine development. Emerg. Microbes Infect..

[101] Wui S.R., Ko A., Ryu J.I., Sim E., Lim S.J., Park S.A., Kim K.S., Kim H., Youn H., Lee N.G. (2021). The Effect of a TLR4 Agonist/Cationic Liposome Adjuvant on Varicella-Zoster Virus Glycoprotein E Vaccine Efficacy: Antigen Presentation, Uptake, and Delivery to Lymph Nodes. Pharmaceutics.

[102] Luan N., Cao H., Wang Y., Lin K., Liu C. (2022). Ionizable Lipid Nanoparticles Enhanced the Synergistic Adjuvant Effect of CpG ODNs and QS21 in a Varicella Zoster Virus Glycoprotein E Subunit Vaccine. Pharmaceutics.

[103] Lee C., Kim M., Chun J., Kim S., Yoon D., Lee H., Bang H., Lee H.J., Park H., Kim Y.B. (2024). Baculovirus Vector-Based Varicella-Zoster Virus Vaccine as a Promising Alternative with Enhanced Safety and Therapeutic Functions. Vaccines.

[104] Garcia-Valcarcel M., Fowler W.J., Harper D.R., Jeffries D.J., Layton G.T. (1997). Induction of neutralizing antibody and T-cell responses to varicella-zoster virus (VZV) using Ty-virus-like particles carrying fragments of glycoprotein E (gE). Vaccine.

[105] Zhu R., Liu J., Chen C., Ye X., Xu L., Wang W., Zhao Q., Zhu H., Cheng T., Xia N. (2016). A highly conserved epitope-vaccine candidate against varicella-zoster virus induces neutralizing antibodies in mice. Vaccine.

[106] Wang H., Zhang S., Xue W., Zeng Y., Liu L., Cui L., Liu H., Zhang Y., Chen L., Nie M. (2024). Glycoprotein E-Displaying Nanoparticles Induce Robust Neutralizing Antibodies and T-Cell Response against Varicella Zoster Virus. Int. J. Mol. Sci..

[107] Levin M.J., Dahl K.M., Weinberg A., Giller R., Patel A., Krause P.R. (2003). Development of resistance to acyclovir during chronic infection with the Oka vaccine strain of varicella-zoster virus, in an immunosuppressed child. J. Infect. Dis..

[108] Levy O., Orange J.S., Hibberd P., Steinberg S., LaRussa P., Weinberg A., Wilson S.B., Shaulov A., Fleisher G., Geha R.S. (2003). Disseminated varicella infection due to the vaccine strain of varicella-zoster virus, in a patient with a novel deficiency in natural killer T cells. J. Infect. Dis..

[109] Leung J., Siegel S., Jones J.F., Schulte C., Blog D., Schmid D.S., Bialek S.R., Marin M. (2014). Fatal varicella due to the vaccine-strain varicella-zoster virus. Hum. Vaccin. Immunother..

[110] Bhalla P., Forrest G.N., Gershon M., Zhou Y., Chen J., LaRussa P., Steinberg S., Gershon A.A. (2015). Disseminated, persistent, and fatal infection due to the vaccine strain of varicella-zoster virus in an adult following stem cell transplantation. Clin. Infect. Dis..

[111] Stadtmauer E.A., Sullivan K.M., Marty F.M., Dadwal S.S., Papanicolaou G.A., Shea T.C., Mossad S.B., Andreadis C., Young J.A., Buadi F.K. (2014). A phase 1/2 study of an adjuvanted varicella-zoster virus subunit vaccine in autologous hematopoietic cell transplant recipients. Blood.

[112] Atiyat R., Elias S., Kiwan C., Shaaban H.S., Slim J. (2021). Varicella-Zoster Virus Reactivation in AIDS Patient After Pfizer-BioNTech COVID-19 Vaccine. Cureus.

[113] Santovito L.S., Pinna G. (2021). A case of reactivation of varicella-zoster virus after BNT162b2 vaccine second dose?. Inflamm. Res..

[114] Maldonado M.D., Romero-Aibar J. (2021). The Pfizer-BNT162b2 mRNA-based vaccine against SARS-CoV-2 may be responsible for awakening the latency of herpes varicella-zoster virus. Brain Behav. Immun. Health.

[115] Chong C.H., Liu C.E., Leong Y.Y., Liao S.Y., Lai H.W., Lee Y.L. (2023). Seroprevalence of varicella-zoster virus antibody and immunogenicity of live attenuated varicella vaccine in healthcare workers in Taiwan. J. Microbiol. Immunol. Infect..

[116] Irwin M.R., Levin M.J., Laudenslager M.L., Olmstead R., Lucko A., Lang N., Carrillo C., Stanley H.A., Caulfield M.J., Weinberg A. (2013). Varicella zoster virus-specific immune responses to a herpes zoster vaccine in elderly recipients with major depression and the impact of antidepressant medications. Clin. Infect. Dis..

[117] Perciani C.T., Sekhon M., Hundal S., Farah B., Ostrowski M.A., Anzala A.O., McKinnon L.R., Jaoko W., MacDonald K.S. (2018). Live Attenuated Zoster Vaccine Boosts Varicella Zoster Virus (VZV)-Specific Humoral Responses Systemically and at the Cervicovaginal Mucosa of Kenyan VZV-Seropositive Women. J. Infect. Dis..

[118] Pahar B., Gray W., Fahlberg M., Grasperge B., Hunter M., Das A., Mabee C., Aye P.P., Schiro F., Hensley K. (2022). Recombinant Simian Varicella Virus-Simian Immunodeficiency Virus Vaccine Induces T and B Cell Functions and Provides Partial Protection against Repeated Mucosal SIV Challenges in Rhesus Macaques. Viruses.

[119] Lowe R.S., Keller P.M., Keech B.J., Davison A.J., Whang Y., Morgan A.J., Kieff E., Ellis R.W. (1987). Varicella-zoster virus as a live vector for the expression of foreign genes. Proc. Natl. Acad. Sci. USA.

